# mTORC1 hyperactivation and resultant suppression of macroautophagy contribute to the induction of cardiomyocyte necroptosis by catecholamine surges

**DOI:** 10.14814/phy2.15966

**Published:** 2024-03-05

**Authors:** Mingqi Cai, Penglong Wu, Wei Ni, Dong Huang, Xuejun Wang

**Affiliations:** ^1^ Heart Center Shanghai Sixth People's Hospital Affiliated to Shanghai Jiao Tong University School of Medicine Shanghai China; ^2^ Division of Basic Biomedical Sciences Sanford School of Medicine of the University of South Dakota Vermillion South Dakota USA; ^3^ Department of Cardiology, Xiamen Cardiovascular Hospital of Xiamen University, School of Medicine Xiamen University Xiamen China

**Keywords:** catecholamine, isoproterenol, macroautophagy, mTORC1, necroptosis

## Abstract

Previous studies revealed a controversial role of mechanistic target of rapamycin complex 1 (mTORC1) and mTORC1‐regulated macroautophagy in isoproterenol (ISO)‐induced cardiac injury. Here we investigated the role of mTORC1 and potential underlying mechanisms in ISO‐induced cardiomyocyte necrosis. Two consecutive daily injections of ISO (85 mg/kg, *s.c.*) or vehicle control (CTL) were administered to C57BL/6J mice with or without rapamycin (RAP, 5 mg/kg, *i.p.*) pretreatment. Western blot analyses showed that myocardial mTORC1 signaling and the RIPK1–RIPK3–MLKL necroptotic pathway were activated, mRNA expression analyses revealed downregulation of representative TFEB target genes, and Evan's blue dye uptake assays detected increased cardiomyocyte necrosis in ISO‐treated mice. However, RAP pretreatment prevented or significantly attenuated the ISO‐induced cardiomyocyte necrosis, myocardial inflammation, downregulation of TFEB target genes, and activation of the RIPK1–RIPK3–MLKL pathway. LC3‐II flux assays confirmed the impairment of myocardial autophagic flux in the ISO‐treated mice. In cultured neonatal rat cardiomyocytes, mTORC1 signaling was also activated by ISO, and inhibition of mTORC1 by RAP attenuated ISO‐induced cytotoxicity. These findings suggest that mTORC1 hyperactivation and resultant suppression of macroautophagy play a major role in the induction of cardiomyocyte necroptosis by catecholamine surges, identifying mTORC1 inhibition as a potential strategy to treat heart diseases with catecholamine surges.

## 1 INTRODUCTION

Coordinated activation of the sympathetic nervous system and adrenal medulla causes the release of catecholamines, meditating the fight‐or‐flight response in the body to physical or emotional stress. However, catecholamine surges and β‐adrenergic hyperactivation have been shown to induce cardiac injury (Chen et al., 2017; Pelliccia et al., 2017; Wang et al., 2020; Zhang et al., 2017). During the progression of chronic heart disease, catecholamine surges and resultant excessive β‐adrenergic stimulation are considered to exacerbate cardiac decompensation and maladaptive remodeling, leading to poor prognosis (Cohn et al., 1984; Colucci et al., 2007). Takotsubo cardiomyopathy, also known as broken heart syndrome or stress cardiomyopathy, is highly associated with the hyperactivation of the sympathetic nervous system when undergoing severe emotional or physical stress (Pelliccia et al., 2017; Wang et al., 2020). In addition, the cardiac injury in the patients with pheochromocytoma or paraganglioma is primarily induced by the striking increase of adrenaline and noradrenaline from chromaffin cells of the tumors (Ferreira et al., 2016; Zhang et al., 2017). Therefore, a better understanding of the mechanisms underlying cardiac injury induced by catecholamine surges and β‐adrenergic hyperactivation is expected to help provide potential new therapeutic strategies for treating the related heart disease.

Mechanistic target of rapamycin (mTOR) is an atypical serine/threonine protein kinase of the phosphoinositide 3‐kinase (PI3K)‐related kinase family. mTOR binds different sets of proteins to form two distinct complexes, mTOR complex 1 (mTORC1) and complex 2 mTORC2 (Wullschleger et al., 2006). mTORC2 plays an important role in the cytoskeleton formation, cell growth, and proliferation; mTORC1 is essential in protein synthesis, cell growth and proliferation, cell metabolism, biogenesis of ribosome, and mitochondria (Saxton & Sabatini, 2017). Also, mTORC1 is a main negative regulator of macroautophagy (Kamada et al., 2000; Saxton & Sabatini, 2017; Zhang, 2015). An extremely high embryonic lethality rate was reported in mice with cardiomyocyte‐restricted knockout of *Mtor* (Zhu et al., 2013). In addition, genetic suppression of mTORC1 in adult mice through tamoxifen‐induced cardiomyocyte‐restricted ablation of *Raptor* resulted in lethal dilated cardiomyopathy (Shende et al., 2011). These lines of evidence indicate an essential and indispensable role of mTORC1 in cardiac development and maintaining normal cardiac function. However, there is a growing body of evidence revealing pharmacological inhibition of mTORC1 has potential protective effects on heart disease (Abdellatif et al., 2018; Buss et al., 2009; Di et al., 2012; Gao, Chen, et al., 2020; McMullen et al., 2004; Sciarretta et al., 2014; Shioi et al., 2003). Inhibition of mTORC1 by rapamycin (RAP) or RAP analogues was reported to reduce cardiomyocyte death, limit myocardial infarct size, and alleviate cardiac remodeling in rodents with myocardial infarction (MI) (Buss et al., 2009; Di et al., 2012; Gao, Chen, et al., 2020). In addition, some studies demonstrated that inhibition of mTORC1 had the potential to prevent and reverse pressure overload‐induced cardiac hypertrophy in mice (McMullen et al., 2004; Shioi et al., 2003). Furthermore, activation of macroautophagy by inhibiting mTORC1 signaling has been considered one of the most promising interventions to maintain cardiovascular health during aging (Abdellatif et al., 2018). To date, whether inhibition of mTORC1 can protect against cardiac injury induced by catecholamine surges and β‐adrenergic hyperactivation remains undefined.

Previous studies have demonstrated that Ca^2+^ overload, oxidative stress, interstitial fibrosis, and inflammation are potentially involved in the induction of cardiac injury by catecholamine surges and β‐adrenergic hyperactivation (Du et al., 2021). Cardiomyocyte death is the most prominent pathological feature of cardiac injury induced by catecholamine surges (Haft, 1974). Isoproterenol (ISO), a type of synthetic catecholamine, has been widely used to model acute myocardial injury in research (Feng & Li, 2010; Krenek et al., 2009; Rau et al., 2017). Notably, the limited relevant research focusing on the mTORC1 signaling in ISO‐induced cardiac injury has generated controversial results. Some studies revealed that activation of mTORC1 signaling to suppress the overactivated macroautophagy could attenuate cardiomyocyte apoptosis and further mitigate cardiac hypertrophy and fibrosis induced by ISO (Fan et al., 2019; Liu et al., 2018). On the other hand, Mao et al. (2020) reported that suppression of PI3K/AKT/mTOR signaling attenuated oxidative stress, apoptosis and mitochondrial dysfunction in H9C2 cells treated with ISO, suggesting that activation of mTOR signaling exacerbates cardiac injury induced by ISO. Therefore, further investigation into the role of mTORC1 signaling in ISO‐induced myocardial injury and deciphering whether mTORC1‐regulated macroautophagy is potentially involved are warranted. Additionally, our previous study showed that a large proportion of cardiomyocyte necrosis induced by high doses of ISO was necroptosis mediated by the RIPK1–RIPK3–MLKL pathway (Wu et al., 2021). The effect of mTORC1 in the induction of cardiomyocyte necroptosis by ISO remains unexamined although some reports suggested a potential association between mTORC1 and RIPK1–‐RIPK3–MLKL necroptotic pathway induced by other etiologies (Abe et al., 2019; Liu et al., 2014; Ogasawara et al., 2017; Xie et al., 2020).

To fill the critical gaps identified above, we conducted the present study using the ISO‐induced cardiac injury models in mice and cultured cardiomyocytes. We observed a hyperactivation of mTORC1 in ISO‐treated mouse hearts and cultured cardiomyocytes and discovered a protection from mTORC1 inhibition against ISO‐induced cardiac injury, particularly against ISO‐induced cardiomyocyte necroptosis. This protection was associated with the relief of the downregulation of representative target genes of TFEB in ISO‐treated mice. Collectively, these results indicate that hyperactivation of the mTORC1 signaling and resultant suppression of macroautophagy contribute to the cardiomyocyte necroptosis induced by ISO, identifying inhibition of mTORC1 signaling by, for example, RAP as a potential new strategy to prevent or more effectively treat cardiac injury induced by catecholamine surges and β‐adrenergic hyperactivation.

## 2 MATERIALS AND METHODS

### 2.1 Animals and pharmacological treatment

The care and use of animals in this study were approved by the Institutional Animal Care and Use committee (IACUC) of the University of South Dakota or the IACUC of Xiamen Cardiovascular Hospital of Xiamen Univeristy. Two cohorts of C57BL/6 in‐bred mice of both sexes were used for in vivo experiments. Mice of cohort one were randomly divided into three groups: control (CTL), ISO, and RAP + ISO. Mice were subjected to two consecutive subcutaneous injections of ISO (Isoproterenol hydrochloride, #I6504, Millipore Sigma) with an interval of 24 h between the two injections (85 mg/kg) to induce cardiomyocyte injury as previously reported (Feng & Li, 2010; Wong et al., 2017). The injection of an equivalent amount of vehicle (saline, 5 μL/g of body weight) was used as CTL for ISO. RAP (Rapamycin, #13346, Cayman Chemical Company) dissolved in saline containing 10% DMSO was intraperitoneally administered (5 mg/kg) 30 min before each ISO dose. The injection of 10% DMSO in saline (5 μL/g of body weight) was used as the CTL for RAP. In the CTL group, the CTL for ISO and the CTL for RAP were administered concurrently with ISO or RAP injections. The mice were euthanized using carbon dioxide (CO_2_) at a gas flow rate of 3.0 L/min followed by heart removal 24 h after the second ISO injection. The ventricular myocardium was collected for further biochemistry and histopathology analyses.

### 2.2 Protein extraction and western blot analyses

Total proteins were extracted from ventricular myocardial samples or cultured neonatal rat cardiomyocytes (NRCMs) samples with the lysis buffer containing 41 mM Tris–HCl, 1.2% sodium dodecyl sulfate (SDS), and 8.2% glycerol. The Halt™ Protease and Phosphatase Inhibitor Cocktail (#78441, ThermoFisher Scientific) was added into the lysis buffer (1:100) to inhibit protein degradation and dephosphorylation. The protein concentration of samples was determined using bicinchoninic acid (BCA) reagents (#23222, #23224, ThermoFisher Scientific) and the absorbance of the protein samples was measured using a spectrophotometer (VICTOR Nivo™, PerkinElmer) at a wavelength of 562 nm. Equal amounts of proteins were fractionated with 8%–14% SDS polyacrylamide gel electrophoresis (SDS‐PAGE) and transferred onto a polyvinylidene difluoride (PVDF) membrane (#162‐0177, Bio‐Rad, Hercules, CA) using a transblot apparatus (Bio‐Rad). The PVDF membrane carrying the proteins transferred was then blocked with 2.5% bovine serum albumin (BSA) in Tris‐buffered saline containing 0.2% Tween 20 (TBST) for 1 h at room temperature to prevent antibodies from nonspecific binding before incubated with the following primary antibodies overnight at 4°C: anti‐LC3 (#2775S, 1:1000, Cell Signaling Technology), anti‐mTOR (#2983S, 1:500, Cell Signaling Technology), anti‐p(Ser2481)‐mTOR (#2974S, 1:500, Cell Signaling Technology), anti‐P70S6K (#2708S, 1:500, Cell Signaling Technology), anti‐p(Thr389)‐P70S6K (#9205S, 1:500, Cell Signaling Technology), anti‐4EBP1 (#9644S, 1:1000, Cell Signaling Technology), anti‐p(Thr31/46)‐4EBP1 (#2855S, 1:1000, Cell Signaling Technology), anti‐actin (#2172, 1:1000, Sigma Aldrich), anti‐RIPK1 (#3493, 1:1000, Cell Signaling Technology), anti‐RIPK3 (#14401S, 1:1000, Cell Signaling Technology), anti‐p(Ser345)‐MLKL (#37333S, 1:500, Cell Signaling Technology), anti‐MLKL (#37705S, 1:1000, Cell Signaling Technology), anti‐CD45 (#AF114, 1:500, R&D Systems), or anti‐Galectin 3 (#12733S, 1:500, Cell Signaling Technology). Then, the PVDF membrane was washed with TBST buffer (10 min × 3 times) to remove unbound primary antibodies before being incubated with corresponding horseradish peroxidase (HRP)‐conjugated goat anti‐mouse IgG or goat anti‐rabbit IgG secondary antibodies (#115‐035‐003, #111‐035‐003, 1:10000, Jackson ImmunoResearch Laboratories) for 1 h in room temperature. Then, secondary antibody solution was replaced with TBST buffer to wash off unbound antibodies (10 min × 3 times). The bound secondary antibody signals on the PVDF membrane were detected using the enhanced chemiluminescent reagent (#34580, Pierce™ ECL Western Blotting Substrate, ThermoFisher Scientific) and imaged in ChemiDoc MP imaging system (Bio‐Rad). The digital images were analyzed and quantified with Image Lab™ software. To most SDS‐PAGE gels, 0.5% 2,2,2‐Trichloroethanol (#139445000, ThermoFisher Scientific) was added to enable the stain‐free protein imaging and the in‐lane total protein content derived from this technology was used as loading CTL (L.C.) for quantitative comparisons of western blot image intensities as we recently reported (Yang et al., 2023).

### 2.3 The LC3‐II flux assay

The LC3‐II flux assay was performed as previously reported with minor modifications (Su et al., 2013). In brief, mice of the ISO and the CTL groups were subjected to an intraperitoneal injection of 3 μmol/kg BafA1 (Bafilomycin A1, #11038, Cayman Chemical Company) or vehicle CTL (DMSO in saline) 1 h before the ventricular myocardium was sampled for western blot analyses of LC3‐II. The LC3‐II flux is the difference of LC3‐II protein levels between the samples with or without BafA1 treatment of the same group, referring to the net amount of LC3‐II accumulated by BafA1‐induced inhibition of lysosomal function. The LC3‐II flux assays are considered the “gold standard” method for reflecting autophagic activity (Gottlieb et al., 2015).

### 2.4 RNA isolation and real time reverse transcriptase PCR (qPCR)

Ventricular myocardium samples were collected and stored in RNAlater (#AM7024, ThermoFisher Scientific) in 4°C refrigerator. Three days later, the RNAlater was removed, and the tissue samples were placed in −80°C freezer for long‐term storage before RNA extraction. Total RNA was isolated from the stored myocardial samples using TRI reagent (#TR118, Molecular Research Center) as described before (Zhang et al., 2019). The concentration and quality of RNA was tested using Nanodrop assay (Nanodrop 2000, ThermoFisher Scientific). One μg of isolated RNA was used as a template to generate the first‐strand cDNA using Applied Biosystem™ High‐Capacity cDNA Reverse Transcription Kit (#4368814, ThermoFisher Scientific) following the manufacturer's instructions. For qPCR, 2 μL diluted cDNA samples were used as templates. A master mix was prepared with PowerUp™ SYBR™ Green Master Mix (#25742, ThermoFisher Scientific), primers, and DEPC water. An aliquot of 18 μL master mix and 2 μL cDNA template were transferred to each well of a 96‐well plate. The StepOnePlus real time PCR system (Applied Biosystems, ThermoFisher Scientific) was used. The program was set up as followed: 50°C for 2 min hold, 95°C for 10 min hold, 40 cycles of 95°C for 15 s and 60°C for 60 s. mRNA levels of mouse *Gapdh*, *M6pr*, *Mcoln1*, *p62/Sqstm1*, *Uvrag*, and *Vps18* were measured as reported (Pan et al., 2020), using the following primers:

*Gapdh*, 5′‐ATGACATCAAGAAGGTGGTG‐3′ (forward) and 5′‐CATACCAGGAAATGAGCTTG‐3′ (reverse).

*M6pr*, 5′‐CTCATCTCCCTCTACTGGTACTT‐3′ (forward) and 5′‐CGCTTCCATTCCCTTCCTTTA‐3′ (reverse).

*Mcoln1*, 5′‐CTGTCATCTACCTGGGCTATTG‐3′ (forward) and 5′‐GAGTGAGAACAGACACTCAGAAA‐3′ (reverse).

*p62/Sqstm1*, 5′‐CTCTGGACACGATCCAGTATTC‐3′ (forward) and 5′‐ CTGCTCTACGTGATGCAACTA‐3′ (reverse).

*Uvrag*, 5′‐GACCACGAGACAGTTGAGATAG‐3′ (forward) and 5′‐GCAGGGACAATGGACTTAGAA‐3′ (reverse).

*Vps18*, 5′‐GGACTTGATGGCTTTGTGTTG‐3′ (forward) and 5′‐CGTGACCTGACCTGTTCTATTC‐3′ (reverse).

### 2.5 Evans blue dye uptake assay

Evans blue dye (EBD; #314‐13‐6, Sigma‐Aldrich) dissolved in saline (10 mg/mL) was intraperitoneally injected into the mice at a dose of 100 μg/g body weight 18 h before ventricular myocardium was sampled. The EBD was then absorbed into the circulatory system and bound to albumin. Only when the integrity of the cell membrane is destroyed can EBD enter the cytoplasm of the cell and show red auto‐fluorescence (Su et al., 2011). Therefore, EBD uptake assay was used to detect the integrity of cell membrane to assess the extent of necrosis in cardiomyocytes. Mice were retrogradely perfused with phosphate‐buffered saline (PBS) to flush out blood and EBD in the vasculature and interstitial space under 4% isoflurane. The heart tissue was then perfused and in situ fixed by replacing PBS with 4% paraformaldehyde. The fixed ventricular myocardium was equilibrated with low to high concentrations (10%–30%) of sucrose solution before being embedded in the Tissue‐Tek® (optimal cutting temperature) O.C.T. Compound (Sakura Finetek USA). The myocardium embedded in the Tissue‐Tek® O.C.T. Compound was rapidly frozen and stored at −80°C. Cryosections (7 μm) of ventricular myocardium were counterstained with DAPI (4′,6‐diamidino‐2‐phenylindole) and Alexa Fluor™ 488 conjugated phalloidin to show nuclei (blue) and F‐actin (green) in cardiomyocytes respectively. Imaging with the excitation wavelength at 620 nm and emission wavelength at 680 nm, EBD in the cytoplasm of cardiomyocytes displays red auto‐fluorescence. Leica TCS SP8 STED 3X White Light Laser and Super‐Resolution Confocal Imaging System (Leica Microsystem, Buffalo Grove, IL, USA) were used to image myocardial sections. The percentage of the EBD‐positive area in representative myocardial cryosections from 4 mice (three males and one female) per treatment group was measured by ImagePro Plus 6.0 software.

### 2.6 Isolation, culture, and treatment of NRCMs

Primary NRCMs were isolated from the ventricles of 2‐day‐old Sprague–Dawley rats using the Cellutron Neomyocytes isolation system (#nc‐6031, Neomyt Kit, Cellutron Life Technology) following the manufacturer's instructions. The NRCMs were plated on 6 cm dishes at a density of 2.0 × 10^6^ cells per dish in DMEM (#SH30243.02, Cytiva) with 10% FBS (#EF‐0500‐A, Atlas Biologicals) and 1% antibiotics (Antibiotic‐Antimycotic solution, #30‐004‐CI, Corning) and cultured in a 5% CO_2_ incubator at 37°C as previously described (Pan et al., 2017). The cultured NRCMs were treated with 0, 10 or 100 μM ISO for 30 min before cell lysates were harvested.

### 2.7 LDH assay

Lactate dehydrogenase (LDH) assay was performed as previously described (Pan et al., 2017). LDH activity released from damaged cells is an index of cytotoxicity. LDH leakage in the culture media collected from treated NRCMs was measured using a Cytotoxicity Detection Kit plus (#04744926001, Roche) following the manufacturer's instructions. Collected media (100 μL) was mixed with 100 μL reaction reagent in 96‐well plate for 30 min at 25°C. Duplicate wells were used for each sample. Absorbance of the samples at 490 nm was determined using microplate reader (VICTOR Nivo™, PerkinElmer).

### 2.8 Statistical analyses

The GraphPad Prism (Version 9.5.0; GraphPad Software, Inc., La Jolla, CA) was used for statistical analyses. All data were examined for normality using the Shapiro–Wilk test and homogeneity of variance using Brown‐Forsythe test. All continuous variables were presented as scatter dot plots with mean ± SD superimposed. Differences between two groups were evaluated with two‐tailed unpaired Student's *t* test. Welch's correction was used when the variances in each group were unequal. One‐way ANOVA followed by Tukey's multiple comparisons tests was used to compare the differences among three or more groups. When there were two grouping factors, two‐way ANOVA followed by Tukey's multiple comparisons tests was used. A *p*‐value or adjusted *p*‐value <0.05 was considered statistically significant.

## 3 RESULTS

### 3.1 Myocardial mTORC1 signaling is activated in ISO‐treated mice

**FIGURE 1:**
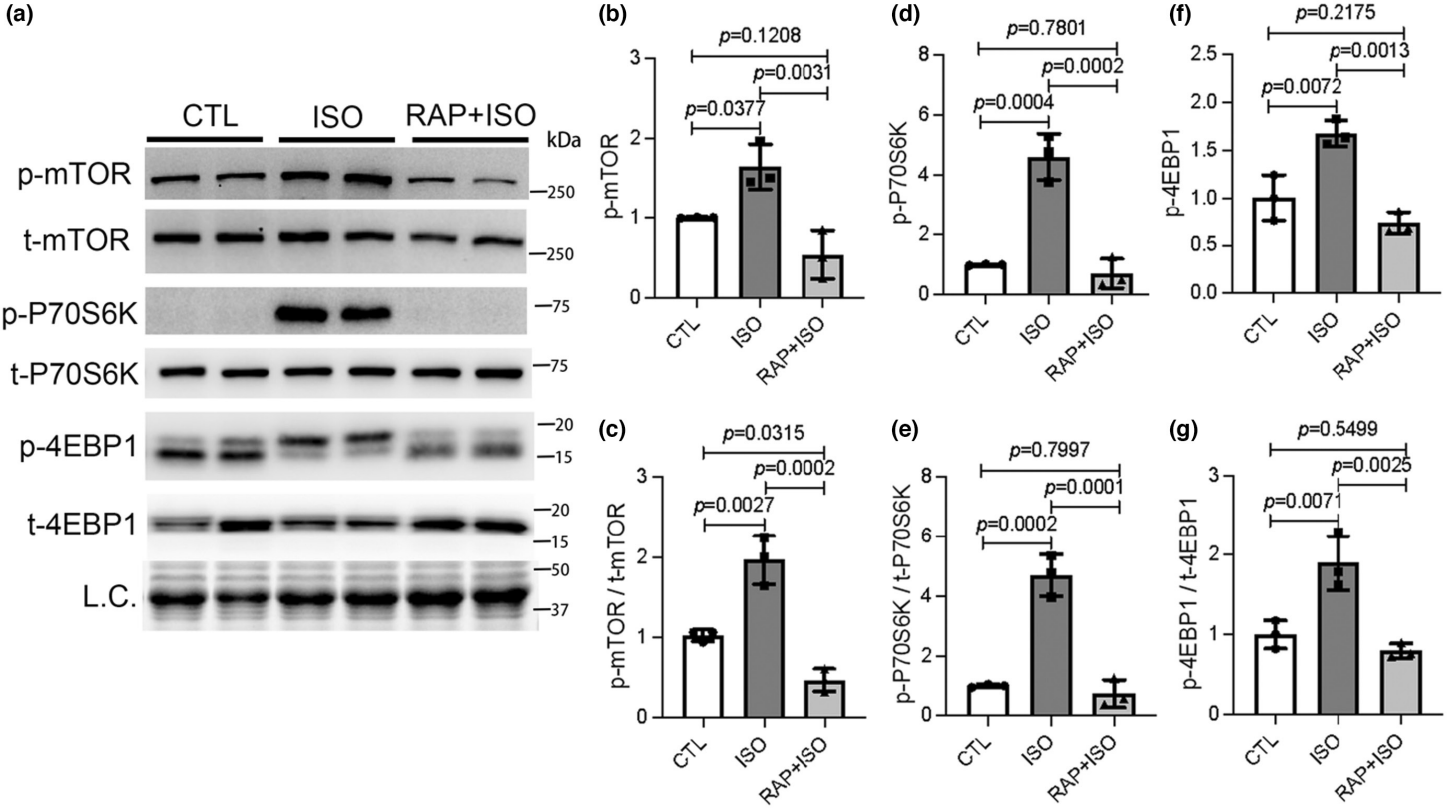
Myocardial mechanistic target of rapamycin complex 1 (mTORC1) signaling is activated in mice treated with isoproterenol (ISO). Mice of the control (CTL), ISO, and rapamycin (RAP) + ISO groups were treated as described in the Method section. Ventricular myocardial samples were collected 24 h after the second injection of ISO and total proteins were extracted for western blot analyses for proteins in the mTORC1 signaling pathway. Representative images (a) and pooled densitometry data (b–g) are shown. t‐mTOR, total mTOR; t‐P70S6K, total P70S6K; t‐4EBP1, total 4EBP1; L.C., loading control, the in‐lane total protein signal from the stain‐free image was used to normalize the loading (the same method is also used in other figures); mean ± SD; *n* = 3 mice per group; one‐way ANOVA followed by Tukey's test.

It remains unclear whether mouse myocardial mTORC1 signaling is affected by ISO treatment. Here, the heart tissues were collected after ISO treatment in WT mice and the total proteins were extracted from ventricular myocardium for protein analyses. Western blot analyses showed that myocardial protein levels of the phosphorylated mTOR (p‐mTOR) as well as two major mTORC1 targets, phosphorylated p70 ribosomal protein S6 kinase (p‐P70S6K) and phosphorylated eukaryotic translation initiation factor 4E‐binding protein 1 (p‐4EBP1), were significantly increased in the ISO treatment group compared to CTL group (Figure 1). And the ratios of phosphorylated to total protein levels of mTOR, P70S6K and 4EBP1 were also significantly increased by ISO treatment compared to CTL, indicating that myocardial mTORC1 signaling is activated in the ISO‐treated mice. As expected, the activation of mTORC1 signaling by ISO treatment was effectively abrogated by RAP pretreatment, which is revealed by that the myocardial protein levels of p‐mTOR, p‐P70S6K, and p‐4EBP1 in the RAP + ISO group not only were significantly lower than those in the ISO group but also returned to the level comparable to or even lower (for p‐mTOR/t‐mTOR ratio) than those of the CTL group (Figure 1).

### 3.2 mTORC1 Inhibition attenuates the ISO‐induced cardiomyocyte necrosis in mice

**FIGURE 2:**
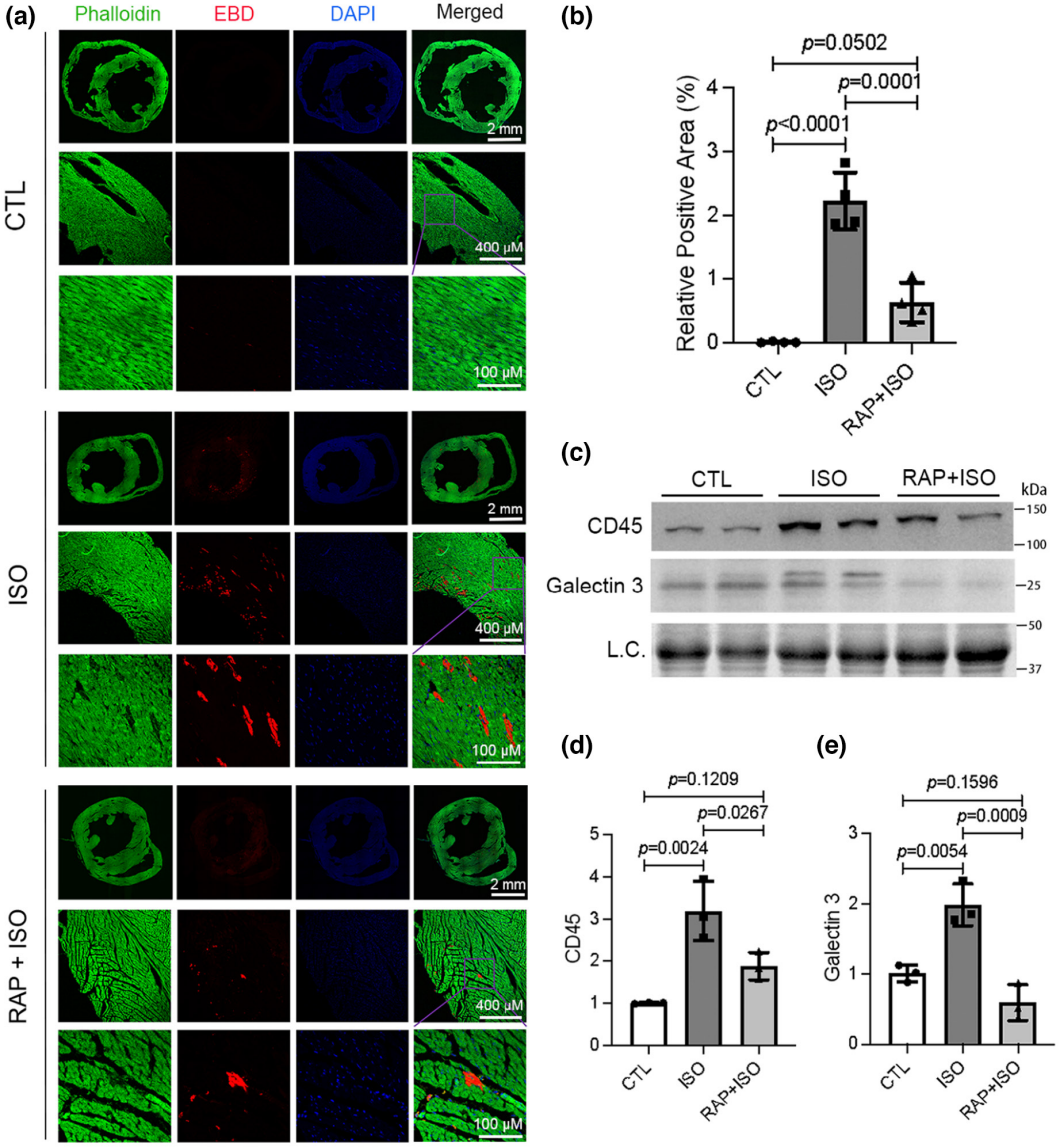
Pretreatment with rapamycin (RAP) attenuates the isoproterenol (ISO)‐induced necrosis in mouse hearts. A and B, Evan's blue dye (EBD) uptake assays. Mice of the control (CTL), ISO, and RAP + ISO groups were subject to an intraperitoneal injection of EBD (100 μg/g, *i.p*.) 6 h after the second saline or ISO injection and sacrificed 18 h later for tissue collection. The cryosections of ventricular myocardium were counterstained with DAPI (blue) and Alexa 488‐conjugated phalloidin (green) before imaged with a confocal microscope. EBD showed red auto‐fluorescence. Shown are representative confocal micrographs taken from anatomically comparable locations (a) and a graph summarizing the percentage EBD‐positive area from 4 mice per group (b). mean ± SD; *n* = 4 mice (3 males and 1 female) per group. (c–e) Representative images (c) and pooled densitometry data (d, e) of western blot analyses for myocardial CD45 and Galectin 3. Total myocardial protein extracts were used L.C., loading control; mean ± SD; *n* = 3 mice per group. *p* Values are derived from one‐way ANOVA followed by Tukey's test.

It is well known that high doses of ISO induce cardiomyocyte necrosis in rodents, which has been used in many studies to model acute MI (Rona et al., 1959a, 1959b; Wong et al., 2017). The pathological hallmark of necrotic cells is the loss of cell membrane integrity and the leakage of cellular content to the extracellular space, which triggers inflammatory responses (Karch & Molkentin, 2015). To determine whether mTORC1 inhibition can mitigate ISO‐induced cardiomyocyte necrosis in mice, we first employed the in vivo EBD uptake assay to assess cardiomyocyte plasma membrane integrity. EBD‐positive cardiomyocytes were readily observed in the ventricular myocardium of mice treated with ISO but not in the mice treated with saline (CTL, Figure 2a,b), indicating that increased cell membrane permeability was induced by ISO. However, this increase in cell membrane permeability was strikingly attenuated by RAP pretreatment as revealed by a significant reduction of EBD‐positive area in the mice treated with RAP + ISO compared to ISO alone, and the difference in EB‐positive area between the CTL and the RAP + ISO groups is not statistically significant (*p* = 0.0502), indicating that RAP pretreatment nearly abolished the ability of ISO to increase cell membrane permeability (Figure 2a,b). Additionally, myocardial protein levels of CD45, a marker of leukocytes, were significantly increased by ISO treatment. Galectin 3 is known to contribute to the initiation and amplification of acute inflammatory responses by recruiting macrophages to injury sites (Bouffette et al., 2023). Myocardial protein levels of Galectin 3 were significantly increased in the ISO group compared with that of the CTL group, indicative of enhanced myocardial inflammation by the ISO treatment. More importantly, the increases of CD45 and Galectin 3 were significantly less in the ISO + RAP group compared with the ISO group (Figure 2c–e), which indicates that RAP alleviated ISO‐induced myocardial inflammation in mice. Taken together, these results demonstrate that inhibition of mTORC1 signaling by the RAP pretreatment can reduce or abolish the induction of cardiomyocyte necrosis in mice by ISO.

### 3.3 Inhibition of mTORC1 blunts ISO‐induced activation of the necroptotic pathway in mouse hearts

**FIGURE 3:**
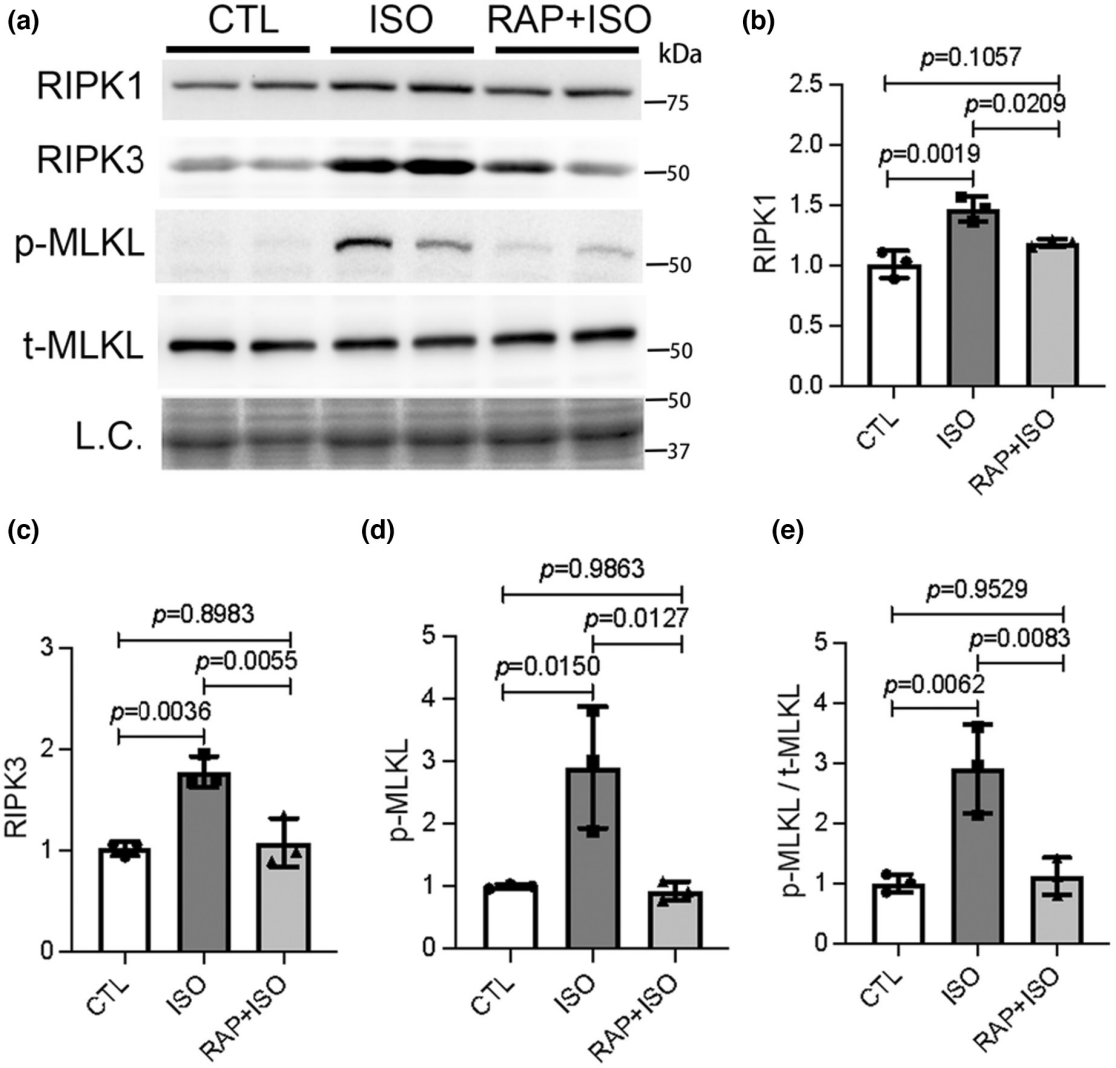
Rapamycin (RAP) pretreatment blunts the activation of the necroptotic pathway by isoproterenol (ISO) in mouse hearts. Total myocardial proteins were extracted from mice in control (CTL), ISO, and RAP + ISO treatment groups and western blot analyses were conducted to detect the protein levels of RIPK1, RIPK3, p‐MLKL, and t‐MLKL. Representative images (a) and pooled densitometry data (b–e) are shown. L.C., loading control; mean ± SD; *n* = 3 mice per group; one‐way ANOVA followed by Tukey's test.

Necroptosis is a form of regulated necrosis that canonically results from activation of the RIPK1–RIPK3–MLKL pathway (Galluzzi et al., 2018). We have previous demonstrated that a large proportion of cardiomyocyte necrosis induced by ISO belongs to necroptosis mediated by the RIPK1–RIPK3–MLKL pathway (Wu et al., 2021). In this study, we sought to explore the role of mTORC1 signaling in the ISO‐induced myocardial injury, particularly in necroptosis. Total myocardial proteins were extracted from mice in CTL, ISO, and RAP + ISO treatment groups. Western blot analyses for RIPK1, RIPK3, and phosphorylated and total MLKL were conducted. Consistent with the previous study, significantly increased protein levels of RIPK1, RIPK3, and phosphorylated MLKL (p‐MLKL) were observed in the ISO treatment group compared with the CTL, indicative of the activation of RIPK1–RIPK3–MLKL‐dependent necroptotic pathway by ISO; however, these increases by ISO were strikingly attenuated by pretreatment with RAP (Figure 3). These results suggest that the activation of necroptotic pathway in the heart by ISO is in part mediated by the mTORC1 signaling pathway.

### 3.4 Inhibition of mTORC1 attenuates ISO‐induced cytotoxicity in cultured cardiomyocytes

**FIGURE 4:**
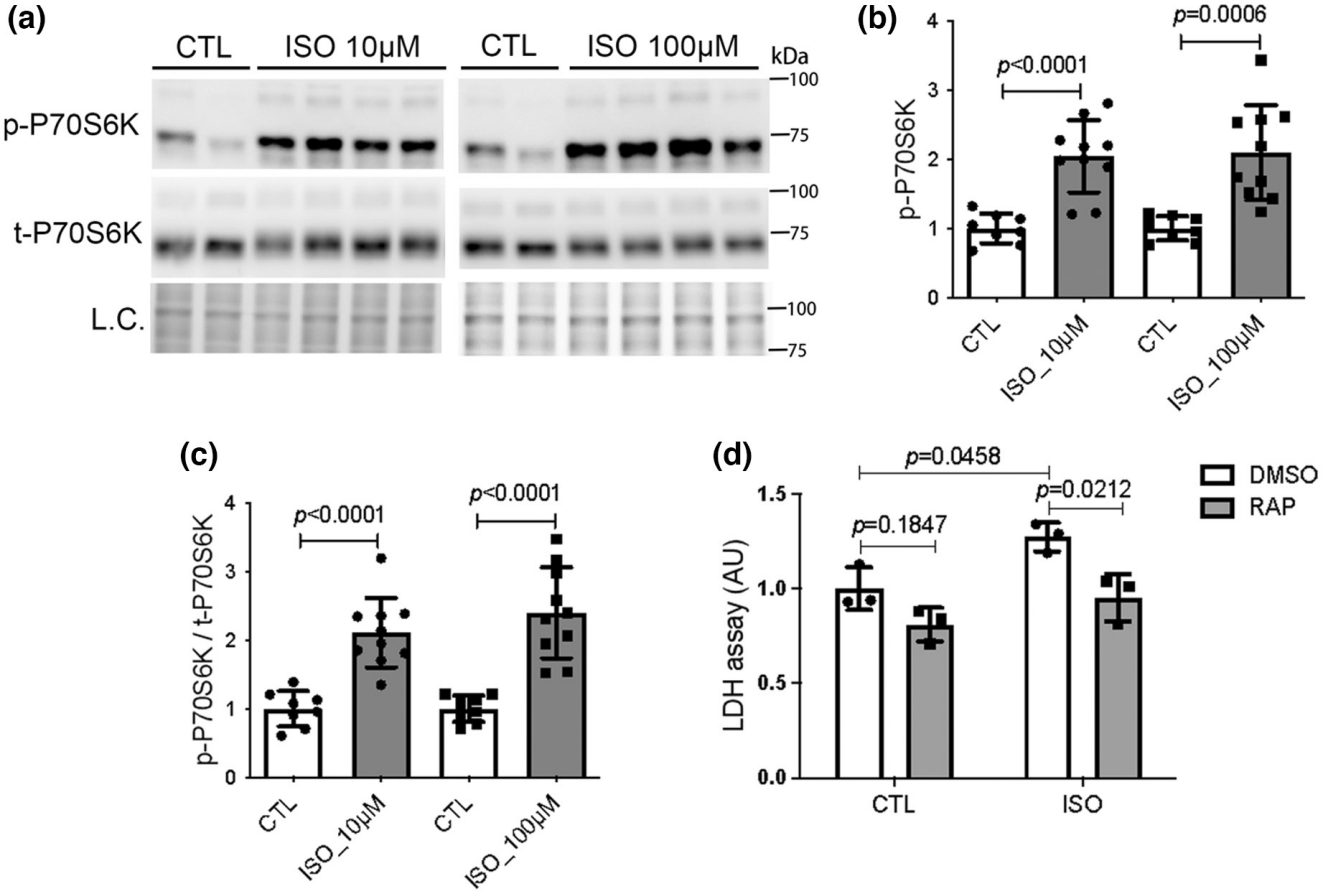
Mechanistic target of rapamycin complex 1 is activated by isoproterenol (ISO) and rapamycin (RAP) treatment attenuates ISO‐induced cytotoxicity in cultured cardiomyocytes. (a–c) Representative images (a) and pooled densitometry data (b, c) of western blot analyses for phosphorylated (p‐) and total (t‐) P70S6K. Cultured neonatal rat cardiomyocytes (NRCMs) were treated with 0, 10, or 100 μM ISO for 30 min before the cells were harvested and lysed to extract total proteins for the analyses. L.C., loading control; mean ± SD; *n* = 8 biological repeats in control (CTL) group and *n* = 10 biological repeats in 10 μM or 100 μM ISO treatment group; Unpaired *t* test with Welch's correction (CTL vs. ISO_10μM, CTL vs. ISO_100μM). (d) Lactate dehydrogenase (LDH) leakage assays. The culture media from NRCM cultures treated with CTL + DMSO, CTL + RAP, ISO + DMSO, or ISO + RAP for 48 h were collected for LDH activity assays. AU, arbitrary unit; mean ± SD; *n* = 3 biological repeats per group; two‐way ANOVA followed by Tukey's test.

To test if the activation of mTORC1 signaling by ISO in mouse hearts could be cardiomyocyte autonomous, we examine the effect of ISO on mTORC1 signaling in cultured NRCMs. The cultured NRCMs were treated with 10 or 100 μM ISO, which are commonly used to induce cytotoxicity (Li et al., 2018; Luan et al., 2015; Rau et al., 2017). The NRCMs treated with the same volume of media served as the CTL group (CTL). The cell lysates were harvested for western blot analyses of the phosphorylation status of mTORC1 downstream target protein P70S6K. Consistent with what we found in mice, both 10 μM and 100 μM ISO treatment induced an upregulation of phosphorylated P70S6K (Figure 4a–c), indicative of mTORC1 activation. Next, we examined whether mTORC1 inhibition attenuated the cytotoxicity induced by ISO. The cell culture dishes were randomly divided into four treatment groups: CTL + DMSO, CTL + RAP, ISO + DMSO, and ISO + RAP. The LDH activity in the culture media was detected to assess cytotoxicity. As anticipated, ISO treatment significantly increased the LDH activity in the media of NRCMs cultures compared to CTL **(**Figure 4d **)**. However, the increased LDH activity was significantly reduced by RAP treatment **(**Figure 4d **)**, suggesting that mTORC1 inhibition by RAP attenuates ISO‐induced cytotoxicity in cultured NRCMs.

### 3.5 ISO‐induced suppression of myocardial TFEB signaling is prevented by inhibition of mTORC1

**FIGURE 5:**
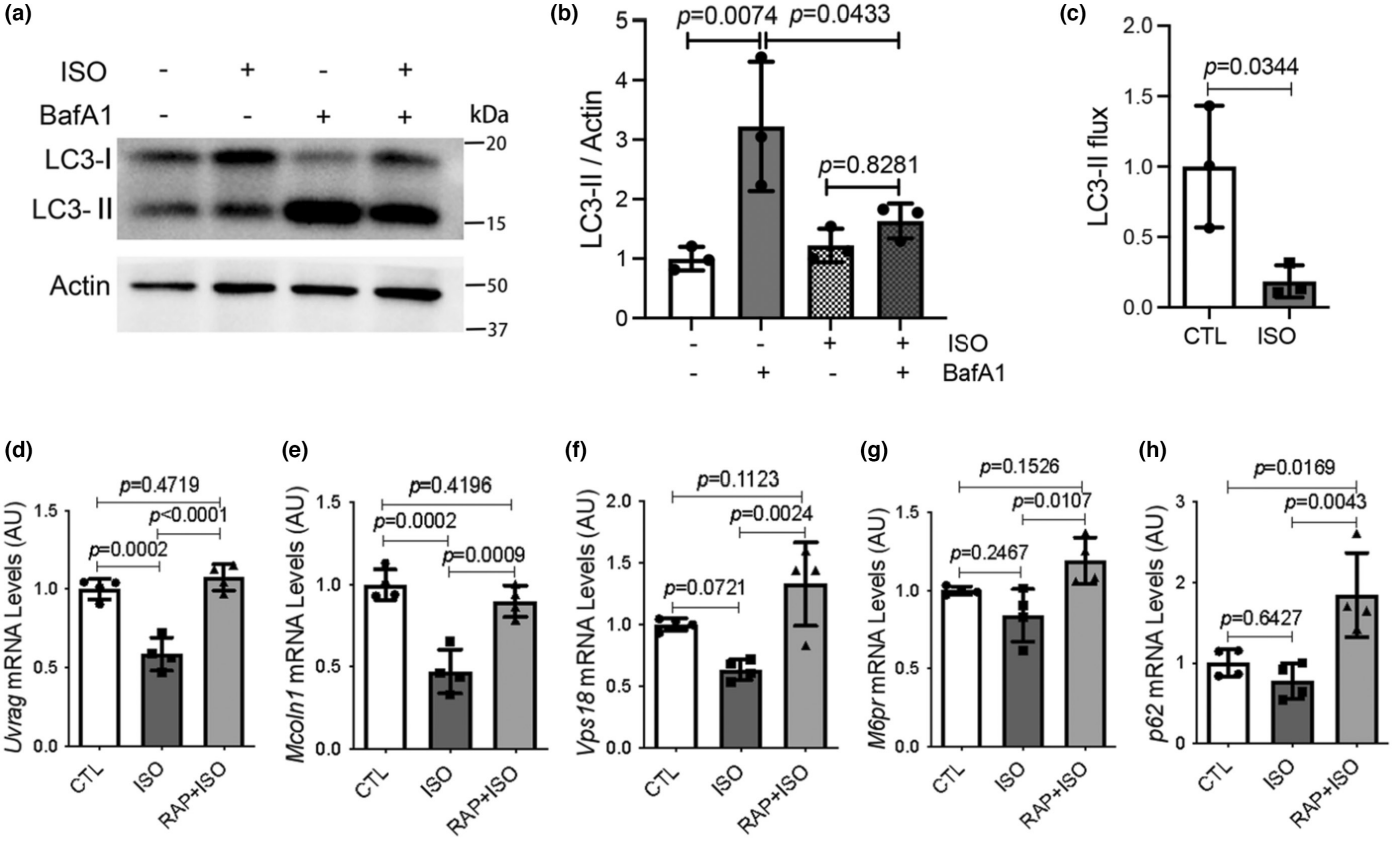
Isoproterenol (ISO) treatment impairs myocardial autophagic flux and rapamycin (RAP) pretreatment prevents ISO from downregulating representative target genes of TFEB in mouse hearts. (a–c) Mice treated with or without ISO were subjected to intraperitoneal injection of 3 μmol/kg bafilomycin‐A1 (BafA1) or same concentration of DMSO in saline as control 1 h before the ventricular myocardium was sampled for western blot analyses of LC3‐II. Actin was probed as loading control. Representative images (a) and pooled densitometry data for LC3‐II (b) are shown. LC3‐II flux is the difference in normalized LC3‐II protein levels between the myocardial samples with or without BafA1 treatment. The LC3‐II flux in control (CTL) and ISO groups are shown in panel c. Mean ± SD; *n* = 3 mice per group; *p*‐values are derived from two‐way ANOVA followed by Tukey's test (b) or unpaired *t* test with Welch's correction (c). (d–h) mRNA levels of representative TFEB target genes. Total RNAs extracted from ventricular myocardium were used for real time RT‐PCR analyses of the indicated TFEB downstream target genes. The mean value of mRNA levels in CTL group was set up as 1 arbitrary unit (AU); mean ± SD; *n* = 4 mice per group; one‐way ANOVA followed by Tukey's test.

Previous reports associated both activation and impairment of macroautophagy with the induction of myocardial injury by ISO (Fan et al., 2019; Li et al., 2020; Liu et al., 2018; Lu et al., 2016). To help dissect the controversy, we assessed changes in myocardial autophagic flux after ISO treatment in mice using the standard LC3‐II flux assay (Gottlieb et al., 2015). The vacuole‐ATPase inhibitor bafilomycin A1 (BafA1) was used for blocking the fusion of lysosomes with autophagic vesicles 1 h before the ventricular myocardium was sampled. The western blot results showed that BafA1 treatment significantly increased the protein levels of LC3‐II in the CTL group, whereas BafA1 failed to increase the LC3‐II protein levels in ISO treatment group (Figure 5a,b), leading to the discernibly reduced myocardial LC3‐II flux in mice treated with ISO (Figure 5c), which reflects that autophagic activity is severely impaired in mouse hearts by the ISO treatment.

Transcription factor EB (TFEB) is a master regulator of lysosomal genesis and the autophagic‐lysosomal pathway (Pan et al., 2020). The downstream target genes of TFEB are known as Coordinated Lysosomal Expression and Regulation (CLEAR) gene network, consisting of genes involved in lysosomal genesis and autophagic activity (Zhu et al., 2021). Hence, we sought to determine the impact of ISO treatment on the expression of representative TFEB target genes and tested the potential mitigating effect of RAP pretreatment. Quantitative polymerase chain reaction (qPCR) analyses of myocardial mRNA revealed that the expression levels of *Uvrag*, *Mcoln1*, and *Vps18* were discernibly lower in the ISO group compared with those in the CTL group, whereas RAP pretreatment blocked them from the downregulation by ISO (Figure 5d–f). Additionally, the mRNA levels of other representative TFEB target genes, such as *M6pr* and *p62*, tended to be lower in the ISO group compared with the CTL although reduction did not achieve statistical significance due to a bigger variation; but importantly their levels in the RAP + ISO group were significantly greater than in the ISO alone group (Figure 5g,h). These data suggest that activation of mTORC1 mediates ISO‐induced TFEB suppression and macroautophagy impairment in mice.

## 4 DISCUSSION

In the present study, we have discovered that mTORC1 signaling is activated by ISO treatment in both mouse hearts and cultured cardiomyocytes and mTORC1 inhibition by RAP protects against ISO‐induced cardiomyocyte necrosis and cytotoxicity, indicating that mTORC1 signaling mediates ISO induction of cardiomyocyte necrosis. We have further demonstrated that myocardial autophagic flux is strikingly suppressed in the ISO‐treated mice and pretreatment with RAP prevents ISO from suppressing TFEB target gene expression and from activating the canonical necroptotic pathway. These new findings provide compelling evidence that activation of mTORC1 and thereby suppression of macroautophagy play a major pathogenic role in the induction of cardiac injury by catecholamine surges and, accordingly, the inhibition of mTORC1 with RAP, for example, represents a potentially new therapeutic strategy for treating heart diseases related to catecholamine surges and β‐adrenergic hyperactivation.

Both β1‐ and β2‐adrenergic receptors are expressed in mammalian hearts. Activation of β‐ARs play an important role in the regulation of cardiovascular function, including positive inotropic and chronotropic effects. When β‐ARs are stimulated, typically by epinephrine or norepinephrine, the canonical Gαs‐adenylate cyclase (AC)‐cyclic AMP (cAMP)‐protein kinase A (PKA) signaling cascade is activated and further induces downstream signaling pathways (Chia et al., 2019). PKA activation due to loss of PKA the regulatory subunit 1α has been found to increase cardiomyocyte necrosis (Liu et al., 2021).There is an emerging role of cyclic nucleotides and cyclic nucleotide‐dependent protein kinases on the regulation of mTOR signaling (Shi & Collins, 2023), particularly the important role of cAMP/PKA in mTOR activation. The activation of the AKT/mTORC1 pathway in a mouse model of pressure overload cardiac hypertrophy was abolished by the transgenic inhibition of PKA (Bai et al., 2024). Lin et al. has revealed that adrenaline potentiates insulin‐stimulated activation of AKT and mTORC1 downstream signaling via cAMP and the exchange protein directly activated by cAMP (EPAC) in soleus muscles (Brennesvik et al., 2005). Kim et al. have suggested cAMP phosphodiesterase 4D (PDE4D) as a binding partner of Ras homolog enriched in the brain (Rheb) that acts as a cAMP‐specific negative regulator of mTORC1 in HEK293 cells. They found that PDE4D could bind Rheb in a noncatalytic manner and thereby inhibit the ability of Rheb to activate mTORC1. However, elevated cAMP levels can disrupt the interaction of PDE4D with Rheb and thereby induce the activation of mTORC1 signaling (Kim et al., 2010). In adipocytes and mouse adipose tissue, a β‐AR agonist was reported to induce PKA‐mediated phosphorylation of mTOR and Regulatory‐Associated Protein of mTOR (RAPTOR) in mTORC1 and thereby activate mTORC1 to promote fat browning (Liu et al., 2016). These results suggest that the activation of mTORC1 signaling is potentially associated with increased cAMP/PKA by β‐AR stimulation. Additionally, it was shown that β‐AR stimulation can also inhibit autophagy via PKA‐mediated phosphorylation of autophagy‐related protein LC3 (Kroemer et al., 1997). In the early stage of heart failure, mTORC1 overactivation also plays an important role in β‐AR‐mediated inhibition of autophagy (Wang et al., 2015). ISO is a type of synthetic catecholamine and a nonselective β‐AR agonist. In the present study, we speculate that ISO stimulates β‐ARs of cardiomyocytes, which in turn activate the Gαs–AC–cAMP–PKA signaling cascade and then interact with multiple regulators of mTORC1 signaling, thereby inducing mTORC1 activation. Besides, both cAMP/PKA signaling and mTORC1 overactivation could contribute to the myocardial autophagy impairment we observed in the mice treated with ISO. Nevertheless, the specific mechanisms involved in ISO‐induced mTORC1 activation remain to be discovered.

Previous studies addressed the importance of mTORC1 and mTORC1‐regulated macroautophagy in cardiac hypertrophy, myocardial ischemic injury, and heart failure although the role of mTORC1 in the induction of cardiomyocyte necrosis has been controversial. Most studies have acknowledged that mTORC1 signaling is activated in the induction of cardiac hypertrophy by chronic infusion of ISO, probably due to the important regulatory role of mTORC1‐P70S6K in protein synthesis required for cardiac hypertrophy (Bi et al., 2022; Gao et al., 2020a, 2020b; Lin et al., 2022; Mao et al., 2020; Sun et al., 2021). Mao et al. (2020) showed that PI3K–AKT–mTOR signaling was overactivated in a rat Takotsubo Syndrome (TTS) model established by ISO, implying the potential role of PI3K–AKT–mTOR signaling in the cardiac dysfunction induced by stress; they attributed the protection of chronic AKT inhibition against cardiac dysfunction of TTS rats to reduction of mitochondrial reactive oxygen species (ROS) and ROS‐induced apoptosis. As mentioned earlier, the induction of cardiomyocyte necrosis by high dosage of ISO has been frequently utilized to model acute MI (Rona et al., 1959a, 1959b; Wong et al., 2017). However, there is a prominent controversy over the role of mTOR signaling in ISO‐induced MI. Hou et al. (2022) reported that uncoupling protein 1 knockout aggravated ISO‐induced acute MI by inhibiting the AMPK‐mTOR‐PPARα pathway, suggesting that the activated AMPK and subsequent mTOR inhibition by ISO is compensatory in the ISO‐induced MI. Additionally, mTORC1 inhibition by RAP or RAP analogues has been reported to alleviate MI injury in rodents, including the reduction of cardiomyocyte death, decreases in myocardial infarct size and mitigation in cardiac remodeling (Buss et al., 2009; Di et al., 2012; Gao, Chen, et al., 2020). On the other hand, other studies showed that activation of AMPK or mTOR inhibition would activate excessive autophagy and triggered autophagy‐associated cell death and thereby exacerbate ISO‐induced MI. In this situation, activation of mTOR signaling to suppress excessive autophagy was implicated as the potential therapeutic strategy to treat ISO‐induce cardiac injury (Chen et al., 2018; Liao et al., 2022).

**FIGURE 6:**
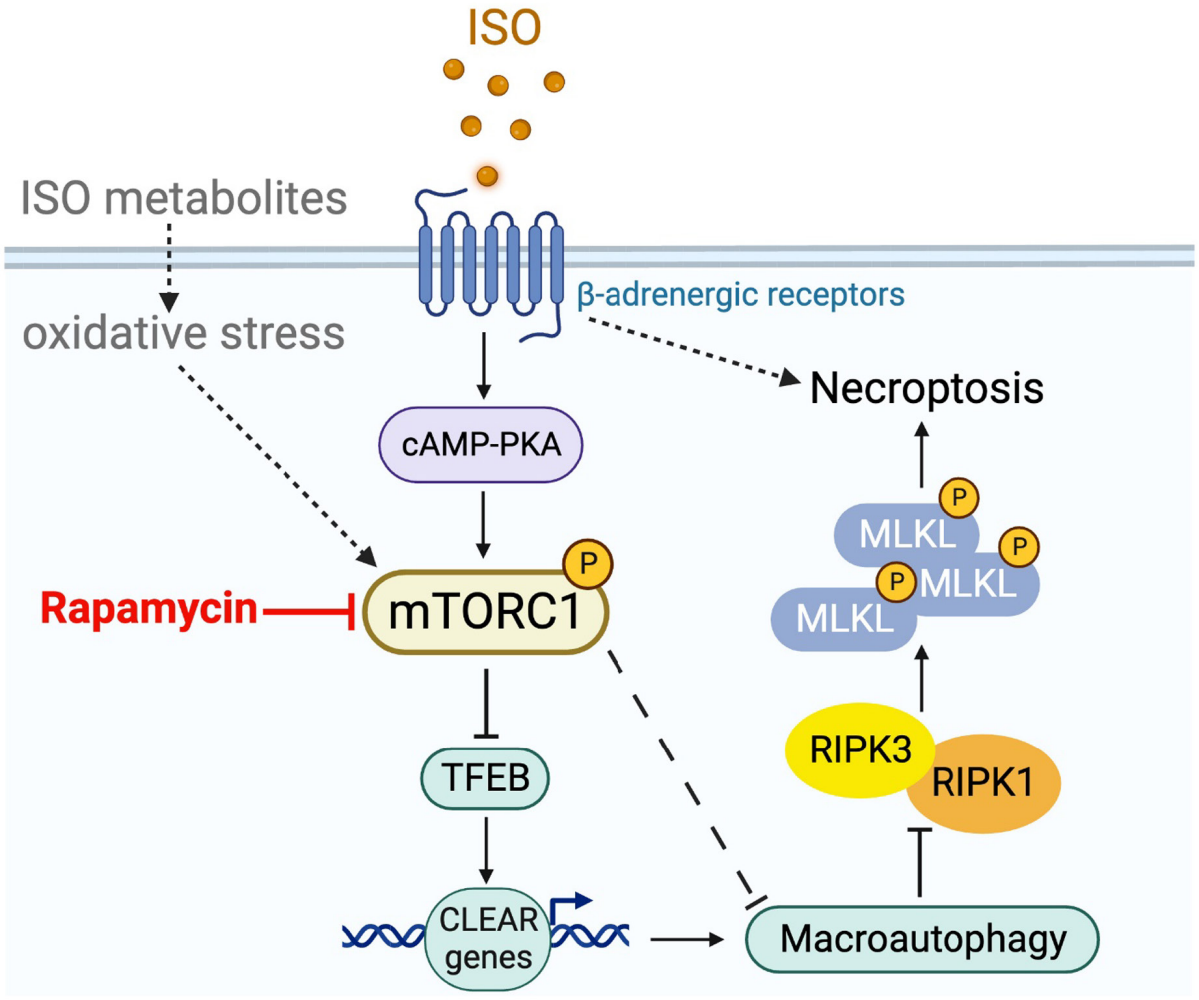
A working model for the role of the mechanistic target of rapamycin complex 1 (mTORC1) and its regulated macroautophagy in the induction of cardiomyocyte necroptosis by large doses of isoproterenol (ISO). It is posited that β‐adrenergic receptor stimulation by ISO activates myocardial mTORC1 signaling, which inhibits macroautophagy in part through suppression of TFEB signaling, which in turn promotes the activation of the necroptotic pathway. Dotted lines depict other potential but not tested pathways.

In the present study, we have unraveled that myocardial mTORC1 signaling is hyperactivated in ISO‐treated mice as reflected by significantly increased protein levels of phosphorylated mTOR and the phosphorylated P70S6K and 4EBP1, two downstream target proteins in mTORC1 signaling (Figure 1). Additionally, impairment of myocardial macroautophagy also occurs in ISO‐treated mice, as evidenced by an apparent decrease in autophagic flux (Figure 5), which is also consistent with an activation of mTORC1 as mTORC1 is known to suppress autophagy at multiple steps (Alers et al., 2012; Kim et al., 2011; Sancak et al., 2010). A further corroboration of myocardial mTORC1 activation and autophagy impairment in ISO‐treated mice comes from the downregulation of representative target genes of TFEB that was prevented by treatment with RAP (Figure 5). This is because TFEB, the master regulator of lysosomal genesis and autophagy, is known to be negatively regulated by mTORC1 (Vega‐Rubin‐de‐Celis et al., 2017; Zhu et al., 2021). Previous studies have revealed that both apoptosis and necrosis are the main modes of cardiomyocyte death induced by ISO. However, the underlying mechanism in which ISO induces cardiomyocyte necrosis is not well studied. Our recent study has shown that a large proportion of cardiomyocyte necrosis induced by ISO belongs to necroptosis and is mediated by a RIPK1–RIPK3‐dependent pathway, the canonical pathway mediating necroptosis (Wu et al., 2021). The results of the present study demonstrate that mTORC1 inhibition strikingly alleviates ISO‐induced activation of the necroptotic pathway as well necrosis in mouse hearts (Figures 2 and 3). This prevention is associated with a significant rescue of the decreases in myocardial expression of representative TFEB target genes (Figure 5). Furthermore, ISO‐induced cytotoxicity in cultured NRCMs was also attenuated by RAP pretreatment (Figure 4). These results demonstrate that mTORC1 inhibition by RAP protects against ISO‐induced cardiomyocyte necroptosis and further provide novel insight into the mechanism underlying the therapeutic benefit of mTORC1 inhibition. Based on these new findings, we propose that through activation of mTORC1, sustained β‐AR stimulation by ISO impairs myocardial macroautophagy via mechanisms likely including inhibition of TFEB, which promotes the activation of the necroptotic pathway, leading to cardiomyocyte necroptosis (Figure 6).

In fact, several prior studies using cell cultures provide support for this proposition. Previous studies seemed to have associated mTORC1 signaling with the necroptotic pathway. Ogasawara et al. treated H9C2 cells with TNF‐α and z‐VAD‐fmk to induce necroptosis. They found that RAP disrupted RIPK1–p62 and RIPK1–‐RIPK3 interactions and partially restored macroautophagy function, thereby mitigated necroptosis (Ogasawara et al., 2017), indicating that mTORC1 might participate in necroptosis. In the hippocampal neuron cell line HT22, TNF‐α, and z‐VAD‐fmk‐induced neuronal necroptosis was accompanied by the activation of the AKT/mTOR pathway. Pharmacologically inhibiting AKT and mTORC1 activity prevented neuronal necroptosis. On the other hand, RIPK1 inhibitor necrostatin‐1 suppressed both RIPK1–RIPK3 pathway and AKT/mTORC1, suggesting an interaction between AKT/mTOR and necroptotic pathways in neurons (Liu et al., 2014). Besides ISO‐induced models, an interplay between macroautophagy and necroptosis has been reported in the cardiac injury induced by other etiologies. Huang et al. have observed the aggregation of autophagosomes and decreased autophagic flux in hypoxic cardiomyocytes. Elevated LC3 was reported to interact with RIPK1 and RIPK3 through the LC3 interacting region (LIR), demonstrating a direct connection between autophagy and necroptotic pathway in the cardiomyocytes and suggesting that dysfunctional autophagy probably triggers cardiomyocyte necroptosis under hypoxia (Huang et al., 2021). Similarly, Qiu et al. (2021) have also indicated that restoration of impaired macroautophagy protects against necroptosis in HL‐1 cells induced by the hypoxia and reoxygenation. Moreover, autophagy and necroptosis are associated with each other in the myocardial ischemic injury. Zhang et al. (2020) reported that impaired macroautophagy triggered RIPK3‐mediated cardiomyocyte necroptosis, consequently exacerbating cardiac dysfunction, and promoting adverse cardiac remodeling following MI. In general, autophagy seems to antagonize necroptosis in cardiomyocytes. Our current study has consistently demonstrated that the pronounced cardiomyocyte necroptosis is accompanied by macroautophagy impairment in mice treated with ISO (Figures 2, 3, and 5). Thus, we believe that the attenuation of ISO‐induced macroautophagy impairment contributes to the protection by RAP against cardiomyocyte necroptosis. Nonetheless, we cannot dismiss the possibility that mTORC1 signaling directly interacts with RIPK1–RPK3–MLKL necroptotic pathway to promote the activation of the necroptotic pathway in cardiomyocytes. Hence, it will be interesting and important for future studies to dissect the potential interplay among mTORC1 signaling, the RIPK1–RIPK3–MLKL necroptotic pathway, and macroautophagy in cardiomyocytes treated with ISO.

## 5 CONCLUSIONS

The evidence presented in this study compellingly supports the main conclusions that the mTORC1 hyperactivation and resultant impairment of macroautophagy contributes to the induction of cardiomyocyte necroptosis by β‐AR overstimulation or catecholamine surges. And the inhibition of mTORC1 signaling by RAP was identified to effectively protect against cardiac injury induced by catecholamine surges and β‐adrenergic hyperactivation, suggesting that mTORC1 signaling can become a potential novel therapeutic target for treating β‐AR overstimulation or catecholamine surges‐induced cardiac injury.

## AUTHOR CONTRIBUTIONS

Conception and design of the study (XW, DH, MC, and PW), data collection and curation (MC, PW, WN), data analyses and manuscript preparation (MC, PW, XW), all authors read and approved the manuscript.

## FUNDING INFORMATION

This work was supported in part by American Heart Association grants 20TPA35490091 (to X.W.) and 23PRE1023108 (to M.C.), as well as by grants from National Natural Science Foundation of China (81871102 and 82172068), the Municipal Science and Technology Commission Medical Innovation Project of Shanghai (20Y11910200), Shanghai Jiaotong University School of Medicine, Two‐Hundred Talent Program as Research Doctor (SBR202204), the Research Physician Program of 3‐year Action Plan for Promoting Clinical Skills and Clinical Innovation in Municipal Hospitals of Shanghai Shen Kang Hospital Development Center (SHD2022CRD039) (to D.H.), and a grant (No. 2023J011682) from Natural Sciences Foundation of Fujian Province, China (to P.W.).

## CONFLICT OF INTEREST STATEMENT

No conflicts of interest, financial or otherwise, are declared by the authors.

## ETHICS STATEMENT

The care and use of animals in this study were approved by the Institutional Animal Care and Use committee (IACUC) of the University of South Dakota or the IACUC of Xiamen Cardiovascular Hospital of Xiamen Univeristy.

## Data Availability

The data supporting this study are available upon reasonable request.
